# Total 25-Hydroxyvitamin D Concentration as a Predictor for All-Cause Death and Cardiovascular Event Risk among Ethnic Chinese Adults: A Cohort Study in a Taiwan Community

**DOI:** 10.1371/journal.pone.0123097

**Published:** 2015-03-25

**Authors:** Kuo-Liong Chien, Hsiu-Ching Hsu, Pei-Chun Chen, Hung-Ju Lin, Ta-Chen Su, Ming-Fong Chen, Yuan-Teh Lee

**Affiliations:** 1 Institute of Epidemiology and Preventive Medicine, College of Public Health, National Taiwan University, Taipei, Taiwan; 2 Department of Internal Medicine, National Taiwan University Hospital, Taipei, Taiwan; 3 Clinical Informatics and Medical Statistics Research Center, Chang Gung University, Taoyuang, Taiwan; Indiana University Richard M. Fairbanks School of Public Health, UNITED STATES

## Abstract

**Background:**

Evidence of an inverse association between serum 25-hydoroxyvitamin D [25(OH)D] and the risk of all-cause death and cardiovascular disease from prospective studies is inconsistent. We tested the relationship between 25(OH)D and the risk among adult ethnic Chinese in Taiwan.

**Methods:**

We conducted a community-based cohort study of 1816 participants (age 60.2±10.2 yrs, 45.0% women) in the Chin-Shan Community Cardiovascular Cohort Study who were free of cardiovascular diseases at baseline and provided 25(OH)D measurements.

**Results:**

During a median 9.6 (interquartile range, 8.8- 10.5) years’ follow-up period, totally 263 cases developed cardiovascular death events and 559 participants were documented to death from any cause. As 25(OH)D concentration increased, the incidence rates of cardiovascular events and all-cause death decreased progressively. 25(OH)D was inversely associated with all-cause death: the adjusted hazard ratio was 0.49 (95% confidence interval [CI], 0.25-0.97) for the third quartile and a significant J-shape relationship was found. The performance measures by integrated discriminative improvement showed significant improvement after adding 25(OH)D information (0.14%, 95% CI, 0.03-0.31, P=0.050, for all-cause death and 0.32%, 95% CI, 0.02-0.62, P=0.018 for cardiovascular events).

**Conclusion:**

These findings suggested a modest inverse association between 25(OH)D and the risk of all-cause death among diabetic participants and a good predictive factor in the community. Further studies to investigate the mechanism of vitamin D role on health effect are warranted.

## Introduction

The worldwide problem for vitamin D deficiency has been proposed to relate to the risks of common cancers and cardiovascular diseases[1–3], and a level of 25-hydroxyvitamin D [25(OH)D] less than 30 ng/mL (75 nmol/L) was considered as the deficient status[3]. However, evidence about the relationship between total 25(OH)D concentration and the risk of all-cause death and cardiovascular events was inconsistent, including inverse association[4,5], and null association[6,7]. This inconsistent findings may be attributed to the study participants with high-risk populations[1]. In addition, the relationship between 25(OH)D concentration and all-cause death and cardiovascular events may be nonlinear in the community. Moreover, the community studies based on ethnic Chinese for the protective role of total 25(OH)D concentration have been scanty. Therefore, we conducted a community-based cohort to investigate the role of total 25(OH)D concentration on the risk of all-cause death and cardiovascular events in Taiwan. In addition, we evaluated the performance ability of 25(OH)D on predicting the risk.

## Materials and Methods

### Study design and participants

Details of this cohort study have been published previously[8–10]. Briefly, the Chin-Shan Community Cardiovascular Cohort (CCCC) Study began in 1990 by recruiting 1703 men and 1899 women aged 35 years old and above, in the Chin-Shan township. A total 1816 participants (50.4%) with available serum 25(OH)D measurements was included into the study. Information about anthropometry, lifestyle, and health conditions was assessed by interview questionnaires in 2-year cycles up to 2011 and the validity and reproducibility of the collected data and measurements have been reported in detail elsewhere[9]. Incident CVD included coronary heart disease and stroke cases. Incident coronary heart disease cases were defined as fatal coronary heart disease and hospitalization due to nonfatal myocardial infarction or percutaneous coronary intervention and coronary bypass surgery. Non-fatal myocardial infarction and hospitalizations for coronary intervention and bypass graft surgery was ascertained by the combined information from patient interviews and medical record review. Incident stroke cases were ascertained according to the following criteria: a sudden neurological deficit of vascular origin that lasted longer than 24 hours, with supporting evidence from the image study and medical records. All-cause deaths were identified from the official certificate documents, further verified by house-to-house visits. The National Taiwan University Hospital Committee Review Board approved the study protocol.

### Laboratory measurements

We performed the biochemical measurements and fatty acid profiles once in the baseline. The procedures involved in blood sample collection were previously reported.[11] Briefly, all venous blood samples were drawn after a 12-hour overnight fast, immediately refrigerated, and transported within 6 hours to the National Taiwan University Hospital. Serum samples were stored at -70°C before conducting batch assays to determine the levels of total cholesterol, triglycerides, and high density lipoprotein cholesterol (HDL-C). To ascertain these levels, standard enzymatic tests for serum cholesterol and triglycerides were employed (Merck 14354 and 14366, Germany, respectively). The supernatants were measured for HDL-C levels following the precipitation of specimens using a reagent of magnesium chloride phosphotungstate (Merck 14993). Low density lipoprotein concentrations were calculated as total cholesterol minus cholesterol in the supernatant obtained through precipitation (Merck 14992).[12]

The serum 25(OH)D levels were evaluated in the hospital laboratory using the 25(OH)-Vitamin D direct Elisa Kit (Immunodiagnostik; Bensheim, Germany), based on a competitive ELISA technique with a selected monoclonal antibody that recognizes 25(OH)D. The results were expressed, after point-to-point calculation, as nmol/L (with 1 ng/mL being equivalent to 2.5 nmol/L)[13], and the inter- and intra-assay CV was 7.0%,

### Statistical analysis

Participants were categorized on the basis of 25(OH)D quartiles; the ANOVA and the chi-square tests were used to compare means and proportions among the quartiles of 25(OH)D concentration. In addition, the age, gender-adjusted partial Spearman correlation coefficients were estimated between 25(OH)D and various atherosclerotic risk factors.

We plotted the Kaplan-Meir survival curves for all-cause death and cardiovascular events according to categories and stratified according to baseline hypertension and diabetes status. The incidence rates of all-cause death and CVD events were calculated by dividing the number of cases by the number of person-years of the follow-up for each quartile. Quartiles of Vitamin D were entered as dummy variables in a Cox proportional hazards regression model to calculate the hazard ratio (HR) and 95% confidence interval (CI), taking the lowest quartile as the reference. Model 1 was adjusted for age groups (35–44, 45–54, 55–64, 65–74, > = 75 years old) and gender only. Model 2 included additional confounding factors: adjusted for the following baseline covariates: body mass index (<18, 18–20.9, 21–22.9, 23–24.9, > = 25 kg/m^2^), smoking (yes/no or abstinence), current alcohol consumption habits (yes/none, defined by > = 1 drink per day), education level (less than 9 years, at least 9 years), occupation (not employed, manual labor, or office job), and regular exercise (yes/no, defined by > = 30 min per day moderate intensity physical activity, such as walking or cycling). Model 3 included additional clinical variables: baseline hypertension (yes/no, defined by baseline systolic blood pressure > = 140 mmHg, diastolic blood pressure > = 90 mmHg, or on hypertensive medication), type 2 diabetes (yes/no, defined as baseline fasting glucose > = 126 mg/dL or on hypoglycemic medications), continuous LDL cholesterol, and HDL cholesterol values. Tests for trend were derived from the regression model with a single term representative of the medians of each quartile. Due to potential high-risk impacts, we performed subgroup analyses based on baseline hypertension and type 2 diabetes. Proportional hazard assumption was not rejected in these Cox models by plotting the log(-log(survival time)) versus log of survival time and including time dependent covariates.

To further investigate the role of 25(OH)D concentration to predict the risk, we compared the model including traditional risk factors and the model adding 25(OH)D and tested the prediction performance using calibration and discrimination ability. First, we assessed the goodness of fit for all models based on the Hosmer-Lemeshow test [14], which was a calibration measure to calculate how close the predicted risks were to the actual observed risks [15], and the results showed the calibration was good. Second, we compared the discrimination ability using the area under receiver operative characteristic curve (AUC). An AUC curve is a graph of sensitivity versus 1-specificity (or false-positive rate) for various cutoff definitions of a positive diagnostic test result [16]. Statistical differences in the AUCs were compared using the method of DeLong et al [17]. The AUC was a global summary measure for discrimination between individuals developing outcomes and those who did not[18]. Third, we compared the models by using the net reclassification improvement (NRI) and integrated discrimination improvement (IDI) statistics [19]. The NRI statistic was based on the reclassification tables and was calculated from a sum of differences between the ‘upward’ movement in categories for event subjects and the ‘downward’ movement in those for nonevent subjects. We presented the NRI according to the *a priori* risk categories of risk. The IDI can be interpreted as a difference between improvement in average sensitivity and any potential increase in average ‘one minus specificity’, and the statistic was a difference in Yates discrimination slopes with and without 25(OH)D information.

In addition, we used the natural cubic splines semi-parametric regression models to examine the relationship between 25(OH)D concentration and the risk of all-cause death and cardiovascular events. Natural cubic splines are smooth polynomial functions that can be used to fit data and accommodate potential changes in the direction of the association between the exposure and the outcome of interests, and are constructed of piecewise third-order polynomials which pass through a set of control points and its linear in this tail beyond the boundary knots[20,21]. A SAS macro named ‘lgtphcurv9’ written by Harvard scholars[22] was used which implements natural cubic spline methodology to fit potential non-linear dose-response curves in proportional hazards models. Likelihood ratio tests were performed to test non-linear and linear relationships.

All statistical tests were two-sided and P values < 0.05 were considered statistically significant. Analyses were performed with SAS version 9.2 (SAS Institute, Cary, NC) and Stata version 11 (Stata Corporation, College Station, Texas).

## Results

A total of 1816 participants (age 60.210.2 yrs, 45.0% women) were free from cardiovascular diseases at baseline and provided serum 25(OH)D measurements were recruited into the study. The participants and non-participants were comparable in baseline characteristics. Table 1 showed the basic characteristics of the study participants according to the 25(OH)D quartiles. Compared with those in the first quartile of 25(OH)D, participants in higher quartiles of 25(OH)D were likely to be younger, to have a lower systolic blood pressure, body mass index, lower total cholesterol, triglycerides, LDL cholesterol, and a higher HDL cholesterol and uric acid level. In addition, fasting and postprandial glucose and insulin level, as well as HOMA, were lower among participants among higher quartiles. C-reactive protein distribution was similar across the quartiles. In addition, participants in the higher quartiles of 25(OH)D levels were likely to be men, current smoker, drinker, and less likely to have hypertension and diabetes status.

**Table 1:** Basic characteristics of the study participants, specified by 25(OH)D concentration.

	Q1		Q2		Q3		Q4		
Range, nmol/L	<39		39.0–50.6		50.6–63.8		> = 63.8		
Number	N = 455		N = 452		N = 455		N = 454		
	Mean	SD	Mean	SD	Mean	SD	Mean	SD	P
Age, yr	61.6	10.6	59.6	11.4	60.0	10.9	59.7	9.8	0.017
Systolic BP, mmHg	132.0	22.6	128.7	20.7	128.8	21.8	127.1	20.4	0.007
Diastolic BP, mmHg	78.1	11.4	77.6	10.5	78.3	11.8	77.0	10.8	0.28
BMI, kg/m^2^	23.8	3.4	23.5	3.4	23.2	3.4	22.8	3.0	<.0001
Total cholesterol, mg/dL	206.2	47.2	205.0	43.8	204.7	48.5	195.1	43.7	0.001
Triglycerides, mg/dL,	148.4	111.0	131.1	87.1	128.9	105.1	114.0	79.7	<.0001
HDL-cholesterol, mg/dL	45.7	13.9	47.0	13.3	46.9	12.6	49.3	12.8	0.001
LDL-cholesterol, mg/dL	147.9	45.5	145.3	42.1	143.6	46.9	132.9	42.9	<.0001
Non-HDL-cholesterol, mg/dL	160.3	47.2	156.9	42.9	157.4	48.6	144.9	44.0	<.0001
Uric acid, mg/dL,	5.63	1.73	5.68	1.69	5.96	1.75	6.05	1.68	0.000
Waist circumference, cm,	85.0	10.9	84.5	10.4	83.7	9.9	82.1	9.0	0.002
Fasting glucose, mg/dL	114.7	36.9	116.7	44.0	111.0	27.8	107.6	26.2	0.0004
HOMA index	2.48	3.29	2.24	2.59	2.32	7.29	1.46	1.95	0.017
Fasting insulin, IU/mL	8.29	9.32	6.96	5.88	7.30	17.53	5.02	5.43	0.002
Postprandial insulin, IU/mL	41.5	48.6	39.1	55.9	34.6	48.4	22.4	23.9	<.0001
C-reactive protein, mg/dL	0.28	0.51	0.30	0.52	0.22	0.30	0.32	0.67	0.26
	N	%	N	%	N	%	N	%	
Gender									<.0001
Men	158	34.7	207	45.8	272	59.8	361	79.5	
Women	297	65.3	245	54.2	183	40.2	93	20.5	
Current smoker	133	29.2	160	35.4	212	46.6	279	61.5	<.0001
Alcohol drinker	95	20.9	115	25.4	157	34.5	217	47.8	<.0001
Job									<.0001
No job	306	67.3	271	60.0	244	53.6	190	41.9	
Labor	88	19.3	112	24.8	163	35.8	230	50.7	
Management/official	61	13.4	69	15.3	48	10.6	34	7.5	
Regular exercise	86	18.9	71	15.7	90	19.8	56	12.3	0.01
Family history of cardiovascular disease	46	10.1	41	9.1	35	7.7	33	7.3	0.40
Hypertension	206	45.4	156	34.6	151	33.3	140	30.9	<.0001
Diabetes mellitus	86	19.0	80	17.9	65	14.5	53	11.7	0.01

Abbreviation: Q, quartile; SD, standard deviation; BP, blood pressure; HDL, high density lipoprotein; LDL, low density lipoprotein; HOMA, homeostasis model assessment; ANOVA and the chi-square tests were used to compare the means and proportions among quartiles.

Table 2 showed the age, gender-adjusted partial Spearman correlation coefficients showed that significant associations between 25(OH)D and various atherosclerotic risk factors, ranging from -0.136 (*P*<.0001) for fasting insulin, -0.126 (<.0001) for triglycerides, -0.109 (*P*<.0001) for waist, -0.049 (<.0001) for LDL cholesterol, to 0.142(*P*<.0001) for HDL cholesterol.

**Table 2:** Partial Spearman correlation coefficients of 25(OH)D and various atherosclerotic risk factor profiles in the study participants.

	Coefficient	P
Systolic blood pressure	-0.006	0.79
Diastolic blood pressure	-0.004	0.86
Body mass index	-0.089	0.0002
Total Cholesterol	-0.003	0.90
Triglycerides	-0.126	<.0001
HDL-cholesterol	0.142	<.0001
LDL-cholesterol	-0.049	<.0001
Non-HDL cholesterol	-0.047	0.051
Uric acid	-0.035	0.14
Glucose	-0.075	0.002
Waist	-0.109	<.0001
HOMA	-0.123	<.0001
C-reactive protein	-0.003	0.93
Fasting insulin	-0.136	<.0001
Postprandial insulin	-0.134	<.0001

All were age and gender-adjusted partial Spearman correlation

After a median 9.6 (interquartile range, 8.8–10.5) years’ follow-up period, totally 263 cases developed cardiovascular death events and 559 participants were documented to death from any cause. Table 3 showed the incidence cases, follow-up person-years, and the rates of cardiovascular disease and all-cause deaths, and the hazard ratios and 95% confidence intervals in the study participants. As 25(OH)D concentration increased, the incidence rates of CVD evens and all-cause death decreased progressively; however, the age and gender-adjusted hazard ratios were not significant for CVD events and all-cause deaths (the adjusted hazard ratios from the lowest to highest quartile were 1, 0.84, 0.85 and 0.75, test for trend, *P* = 0.20 for CVD event, and 1, 1.09, 0.95 and 0.90, test for trend, *P* = 0.38, for all-cause deaths). The patterns of association between 25(OH)D concentration and the risk of coronary heart disease and stroke events was similar to those of all cardiovascular events, yet not reaching significance level (S1 Table).

**Table 3:** The incidence cases, follow-up person-years, and the rates of cardiovascular disease events and all-cause deaths, and the hazard ratios and 95% confidence intervals of 25(OH)D concentrations in the study participants, according to quartiles.

Vitamin D	Q1	Q2			Q3			Q4			
Range, nmol/L	<39	39.0–50.6			50.6–63.8			> = 63.8			
Median, nmol/L	31.9	45.4			56.1			74.8			
**CVD events**											
Cases	80	56			67			60			
Person-year	3192.6	3406.7			3453.4			3441.2			
Rates/1000 py	25.1	16.4			19.4			17.4			
	HR	HR	95% CI		HR	95% CI		HR	95% CI		Trend test
Model 1	1	0.83	0.57	1.20	0.87	0.60	1.25	0.73	0.50	1.08	0.14
Model 2	1	0.84	0.58	1.22	0.86	0.59	1.24	0.74	0.49	1.10	0.15
Model 3	1	0.84	0.57	1.22	0.85	0.58	1.24	0.75	0.49	1.14	0.20
**All-cause death**											
Cases	152	138			139			130			
Person-year	3394.91	3548.93			3610.97			3580.81			
Rates/1000 py	44.8	38.9			38.5			36.3			
	HR	HR	95% CI		HR	95% CI		HR	95% CI		Trend test
Model 1	1	1.04	0.80	1.36	0.98	0.75	1.28	0.94	0.71	1.24	0.58
Model 2	1	1.03	0.79	1.34	0.95	0.73	1.25	0.89	0.67	1.19	0.36
Model 3	1	1.09	0.83	1.42	0.95	0.72	1.25	0.90	0.67	1.21	0.38

Abbreviation: HR, hazard ratio; CI, confidence interval; CVD, cardiovascular disease; py, person year;

Model 1: adjusted for age and gender,

Model 2: Model 1 & additionally adjusted for body mass index, smoking, drinking, marital status, education level, job, and sports activity,

Model 3: Model 2 & additionally adjusted for hypertension, diabetes, LDL cholesterol and HDL cholesterol level,

Cox proportional hazards model was applied for estimating the relative risks and 95% confidence intervals

**Fig 1:**
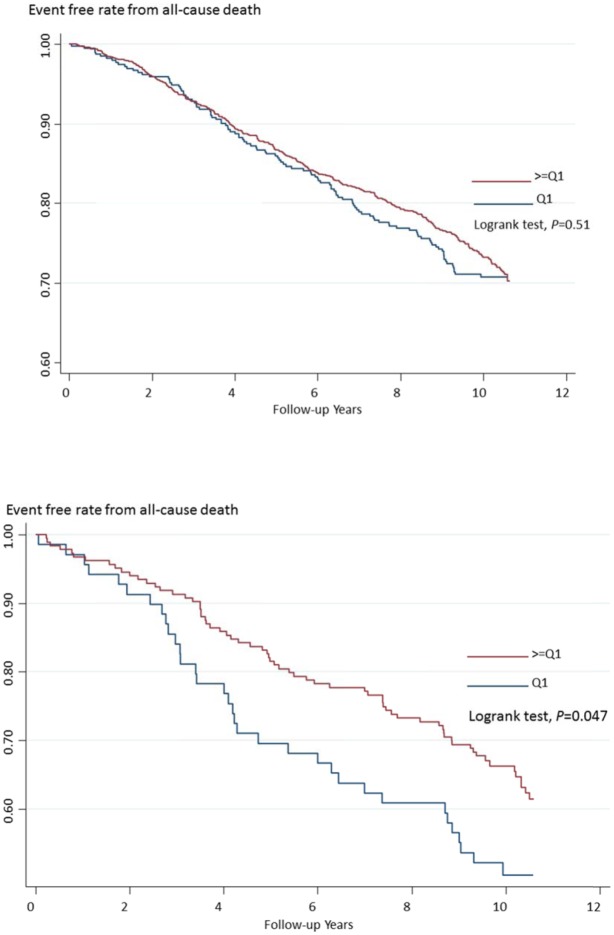
Kaplan-Meir survival curves for the risk of all-cause death in the study participants for all participants (upper) and baseline diabetes status (lower).

In the subgroup analysis according to baseline hypertension and diabetes status (Table 4), we found that 25(OH)D concentration was inversely associated with all-cause death: the adjusted hazard ratio was 0.49 (95% confidence interval [CI], 0.25–0.97) for the third quartile among participants of diabetes. In addition, diabetes played a significant effect modifier for the association between 25(OH)D and the risk of all-cause death (interaction p as 0.023), indicating the protective effect of 25 (OH)D on all-cause death indeed varied by the status of diabetes or not. Fig. 1 showed the Kaplan-Meir curves for the probability of survival free from all-cause death: 25(OH)D was not related to the death in all participants; however, the lowest quartile of Vitamin had a lower survival rate for all-cause death in the participants with diabetes (the log-rank test, *P* = 0.047)

**Table 4:** Subgroup analysis for the adjusted relative risks and 95% confidence intervals of 25(OH)D concentrations in the study participants, according to baseline hypertension and diabetes status.

	Q1	Q2			Q3			Q4				
	HR	HR	95% CI		HR	95% CI		HR	95% CI		P for linear trend*	P for interaction test**
CVD events												
Baseline HT (-)	1	1.01	0.54	1.87	0.97	0.53	1.78	0.69	0.36	1.35	0.23	0.75
Baseline HT (+)	1	0.72	0.44	1.19	0.79	0.47	1.31	0.88	0.50	1.54	0.62	
All-cause death												
Baseline HT (-)	1	1.28	0.86	1.92	1.11	0.74	1.66	0.91	0.59	1.39	0.36	0.46
Baseline HT (+)	1	0.97	0.66	1.43	0.85	0.57	1.28	1.00	0.65	1.55	0.88	
	HR	HR	95% CI		HR	95% CI		HR	95% CI		Trend test	
CVD events												
Baseline DM (-)	1	0.79	0.50	1.26	0.95	0.61	1.46	0.73	0.45	1.17	0.26	0.26
Baseline DM (+)	1	0.97	0.48	1.95	0.68	0.29	1.56	0.98	0.39	2.46	0.75	
All-cause death												
Baseline DM (-)	1	1.17	0.85	1.61	1.14	0.83	1.56	0.90	0.64	1.27	0.44	**0.023**
Baseline DM (+)	1	0.94	0.55	1.60	**0.49**	**0.25**	**0.97**	0.93	0.47	1.83	0.53	

Abbreviation: HR, hazard ratio; CI, confidence interval; CVD, cardiovascular disease; adjusted covariates as Model 3 included age, gender, body mass index, smoking, drinking, marital status, education level, job, sports activity, LDL cholesterol and HDL cholesterol level. Baseline hypertension and diabetes status were used as the stratified factor and not included in each subgroup analysis

* Test for trend with a single term representative of the medians of each quartile

** Interaction test by comparing -2 likelihood ratio values between nested model and full model (adding three dummy variables and diabetes or hypertension status)

With regards to discriminative ability of 25(OH)D to predict the risk of all-cause death and CVD events, the AUCs were not significantly different between the established risk factor models with and without 25(OH)D (S1 Fig.). In addition, the calibration ability indicated by the Hosmer-Lemeshow test showed an acceptable goodness-of-fit (Table 5). Moreover, we found that the performance measures by integrated discriminative improvement (IDI) showed significant improvement after adding 25(OH)D information (IDI = 0.14%, 95% CI, 0.03–0.31, *P* = 0.05, for all-cause death and 0.32%, 95% CI, 0.02–0.62, *P* = 0.018 for CVD event), which was a weak predictive power of 25(OH)D. The subgroup analyses according to baseline diabetes and hypertension status showed consistent improvement of 25(OH)D concentration as the prediction of all-cause death and CVD event risk. We also found that the net reclassification improvement was not statistically significant for the information of 25(OH)D.

**Table 5:** Improvement in discrimination performance and calibration for risk prediction of cardiovascular events and all-cause death in the multivariate-adjusted model after including 25(OH)D concentrations.

		AUC	95% CI		P for HL test	IDI (%)	95% CI		P	NRI (%)	95% CI		P
All-cause death													
Total participants	Established risk factors	0.744	0.719	0.769	4.7	Referent				Referent			
	Established risk factors + Vitamin D	0.744	0.719	0.769	3.1	0.14	0.03	0.31	0.050	-0.30	-2.64	2.05	0.80
Baseline DM (+)	Established risk factors	0.774	0.720	0.828	10.1	Referent				Referent			
	Established risk factors + Vitamin D	0.783	0.729	0.837	4.8	2.30	0.65	3.95	0.003	-1.42	-11.6	8.73	0.79
Baseline DM (-)	Established risk factors	0.739	0.711	0.767	5.8	Referent				Referent			
	Established risk factors + Vitamin D	0.739	0.711	0.767	5.2	0.20	0.02	0.41	0.037	-1.89	-4.83	1.11	0.21
Baseline HT (+)	Established risk factors	0.735	0.696	0.775	7.8	Referent				Referent			
	Established risk factors + Vitamin D	0.736	0.697	0.776	8.0	0.38	-0.11	0.87	0.06	1.44	-3.21	6.08	0.55
Baseline HT (-)	Established risk factors	0.738	0.704	0.771	6.5	Referent				Referent			
	Established risk factors + Vitamin D	0.739	0.705	0.773	7.6	0.53	0.09	0.96	0.008	3.77	-1.31	8.85	0.15
CVD events													
Total	Established risk factors	0.709	0.677	0.741	12.4	Referent				Referent			
	Established risk factors + Vitamin D	0.712	0.679	0.744	16.1	0.32	0.02	0.62	0.018	2.11	-2.09	6.31	0.32
Baseline DM (+)	Established risk factors	0.703	0.636	0.770	14.6	Referent				Referent			
	Established risk factors + Vitamin D	0.714	0.648	0.780	5.3	0.60	-0.49	1.69	0.14	2.47	-3.91	8.86	0.45
Baseline DM (-)	Established risk factors	0.706	0.669	0.743	9.4	Referent				Referent			
	Established risk factors + Vitamin D	0.707	0.669	0.744	10.0	0.33	0.01	0.66	0.023	0.32	-4.52	5.16	0.90
Baseline HT (+)	Established risk factors	0.641	0.589	0.693	4.3	Referent				Referent			
	Established risk factors + Vitamin D	0.649	0.598	0.700	3.3	0.8	0.1	1.6	0.015	5.2	-1.2	11.6	0.11
Baseline HT (-)	Established risk factors	0.700	0.648	0.752	8.0	Referent				Referent			
	Established risk factors + Vitamin D	0.704	0.652	0.756	8.4	0.29	-0.06	0.65	0.053	4.24	-1.77	10.3	0.17

AUC: area under the receiving operative characteristic curves; CI: confidence interval; HL: Hosmer-Lemeshow Goodness-of-Fit Test; CVD: cardiovascular disease; HT: hypertension; DM: diabetes mellitus; NRI: net reclassification improvement; IDI: integrated discrimination improvement;

Established risk factors included age, sex, smoking status, systolic blood pressure, HDL cholesterol, and LDL cholesterol

**Fig 2:**
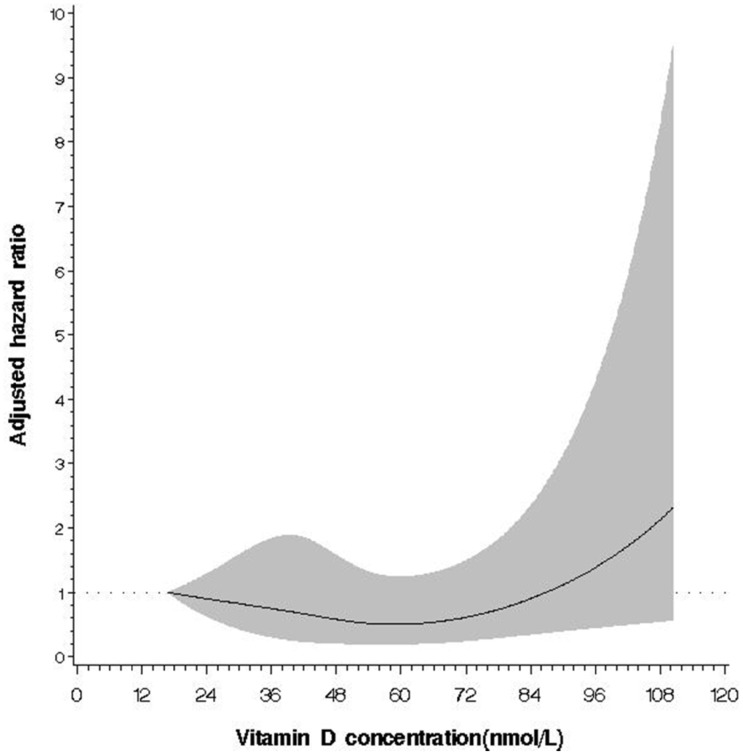
Relationship between 25(OH)D concentration and the risk of all-cause death among the subgroup population of diabetes. The multivariate adjusted hazard ratio is plotted as a function of the 25(OH)D value with the 95% confidence bands shown as the shaded areas (test for nonlinear relation, P = 0.031; test for overall significance of the curve, P = 0.07).

A significant J-shape relation between 25(OH)D concentration and the risk of all-cause death was observed among the diabetic participants (P for non-linear trend = 0.031, Fig. 2), with a null linear association (P for linear trend = 0.07).

## Discussion

In this community-based cohort study, we clearly demonstrated that although 25(OH)D was not related to the risk of all-cause death and CVD events, 25(OH)D indeed was a protective factor for all-cause death among diabetic participants and the relationship between 25(OH)D and all-cause death was not linear.

### Previous literature and current study comparison

Evidence for an inverse association between vitamin D and all-cause death and cardiovascular events was still controversial. Most evidence showed an inverse effect existed for vitamin D as a role for cardiovascular events: Dobnig and colleagues[23] showed that lower 25(OH)D concentration quartiles were related to a higher all-cause death (hazard ratio, 2.08, 95% CI, 1.60–2.70) and cardiovascular death (hazard ratio, 2.22, 95% CI, 1.57–3.13) among high-risk patients. In addition, low Vitamin D concentration (less than 50 nmol/L) was highly prevalent (39%) and was associated with an additional 44% (95% CI, 1.01–2.01, p = 0.04) risk for cardiovascular risk[24] among Danish women. Schierbeck[24] also found that vitamin D deficiency (defined by serum 25(OH)D <50 nmol/L) was associated a 1.49 fold (95% CI, 1.16–1.92) for a composite endpoint, including death, heart failure, myocardial infarction and stroke among Danish women. Moreover, Liu and colleagues showed that 25(OH)D less than 50 nmol/L was associated with a 1.40 fold risk (95% CI, 1.17–1.68) for all-cause death (P<0.001)[5] among adults in USA. Among 18225 men in the Health Professionals Follow-up Study with 10 years of follow up, Giovannucci and colleagues[4] used the 2:1 matched nested case-control study design and showed that lower 25(OH) D concentration (< = 37.5 nmol/L was associated with a 2.42 fold risk (95% CI, 1.53–3.84) for acute myocardial infarction, compared with those of high 25(OH)D (>70 nmol/L), and a graded effect was found[4].

The relationship between vitamin D insufficiency with cardiovascular disease may be more evident among the high-risk group such as hypertension, diabetes mellitus and chronic renal disease[1]. A significant trend for higher risks for all-cause deaths and cardiovascular deaths were found in the lower quartiles among hypertensive participants[25]. In addition, a reverse linear association between 25(OH)D and all cause deaths as well as cardiovascular deaths were found[25], after adjustment for covariates such as blood pressure and medication history.

However, some negative results were available: Jassal and colleagues showed that a higher concentration than mean 25(OH)D concentration as 105 nmol/L did not relate cardiovascular mortality in a community[6]. In addition, vitamin D was a protective factor for cardiovascular mortality only in women, but not men among 3404 adult Swiss population[26]. Moreover, a cohort study based on 312 older Spanish (> = 85 years old) did not showed 25(OH)D was related to cardiovascular death[7]. Our current study showed that overall the association between 25(OH)D and outcomes were not significant; however, the protective effect was found among participants who were diabetic at baseline, and diabetes status had an effect modification to influence the association between 25(OH)D and all-cause death. Our findings implied that the protective effect of 25(OH)D was limited in some specific groups.

### Proposed mechanism of vitamin D for CVD and all-cause death

Our findings supported that in high risk population such as type 2 diabetes, vitamin D indeed provided a protective role for all-cause death. A proposed mechanism for diabetes and cardiovascular disease was through obesity and endothelial dysfunction[27]. Vitamin D regulated macrophage cholesterol metabolism among diabetic patients to facilitate atherosclerosis process[28]. In addition, vitamin D deficiency was accompanied with activation of the renin-angiotensin-aldosterone system[29,30], and the associated increase of parathyroid hormone has been related to insulin resistance[31]. Using the knockout mouse model, Zhou and colleagues[32] clearly demonstrated that Vitamin D was an important regulator for renin-angiotensin system. This modulation of the renin-angiotensin system, in addition to decreasing blood pressure, improved the endothelial function and decreased inflammatory burden, thus limiting progression of atherosclerosis.

### Clinical implication

Literature showed that a proposed 30 ng/mL (75 nmol/L) as the cutoff for vitamin D concentration was sufficient to provide regulatory effects for cardiovascular disease prevention [33]. Regarding with a cross-sectional study based on nation-wide survey in US, Judd and colleagues (2008) found that an optimal 25(OH)D concentration would attenuate the age-associated increase in systolic blood pressure: more than 80 nmol/L was associated with a 20% systolic blood pressure decrease, especially for white population[34]. In addition, seasonable variation of 25(OH)D concentration was observed among 1362 male Health Professionals Follow-up Study[35], and the serum 25(OH)D concentration was estimated as 82 nmol/L in summer and 75 nmol/L in winter among 262 healthy older women living in Taipei[36]. Our study did not provide seasonal variation; however, the sampling time was limited in summer time. The normal range of 25(OH)D was suggested as 50–100 nmol/L (20–40 ng/dL)[37], although some researchers argued that the lowest limit as 5 to 80 nmol/L[38]. Our study showed that a cutoff point at 50 nmol/L provided a discriminative measure for the risk for all-cause death.

### Strengths and weakness

To the best of our knowledge, this is the first prospective cohort study based on ethnic Chinese to investigate the association and prediction of vitamin D and cardiovascular risk and all-cause death. Because of the prospective cohort design in this study, the baseline measurements of participants were not affected by information bias. In addition, the community-based population information could help to reduce the selection bias. Moreover, to control potential confounding factors, we incorporated important socioeconomic and lifestyle factors into the model. We also take consideration into the nonlinear relationship between vitamin D and outcomes. Furthermore, in addition to traditional multivariate adjusted modeling, we applied prediction measures, such as AUCs and integrated discrimination improvement, to demonstrate predictive ability of vitamin D. These measures provided comparative powers for novel biomarker prediction ability[39,40].

However, this study has several potential limitations. First, vitamin D was only once measured and no seasonable variation was explained, the association between vitamin D and outcome risk may be attenuated. Second, we did not examine the supplementary intake and seasonable variations so that we cannot explain the dietary habit role in the risk. Third, due to observational study, residual confounding factors cannot be extensively examined. Finally, potential information bias such as confounding by indication and missing was not completely excluded due to the cohort study design.

## Conclusions

In conclusion, 25(OH)D was inversely associated with the risk of all-cause death among diabetic participants and the relationship may be non-linear among ethnic Chinese adults in a Taiwan community. Further studies to investigate the mechanism of vitamin D role on health effect are warranted.

## Supporting Information

S1 Fig ROC curves for the discriminative performance measures of 25(OH)D concentration for all-cause death (upper) and cardiovascular events (lower) in the study participants. (TIFF)

S1 Table The incidence cases, follow-up person-years, and the rates of specific outcomes, including CAD, all stroke, hemorrhagic and ischemic stroke events, and the hazard ratios and 95% confidence intervals of 25(OH)D concentrations in the study participants, according to quartiles. (DOCX)
