# Supplementary material for: Total 25-Hydroxyvitamin D Concentration as a Predictor for All-Cause Death and Cardiovascular Event Risk among Ethnic Chinese Adults: A Cohort Study in a Taiwan Community
Source: PLoS One. 2015 Mar 25;10(3):e0123097. doi: 10.1371/journal.pone.0123097 (PMC4373875; doi:10.1371/journal.pone.0123097)
Supplement: S1 Table — (DOCX) [file pone.0123097.s002.docx]

**Table S1: The incidence cases, follow-up person-years, and the rates of specific outcomes, including CAD, all stroke, hemorrhagic and ischemic stroke events, and the hazard ratios and 95% confidence intervals of 25(OH)D concentrations in the study participants, according to quartiles**

| 25(OH) D | Q1 | Q2 |  |  | Q3 |  |  | Q4 |  |  |  |
| --- | --- | --- | --- | --- | --- | --- | --- | --- | --- | --- | --- |
| Coronary heart disease | HR | HR | 95% CI |  | HR | 95% CI |  | HR | 95% CI |  | Trend test |
| Model 1 | 1 | 0.92 | 0.50 | 1.67 | 0.68 | 0.36 | 1.29 | 0.74 | 0.39 | 1.39 | 0.28 |
| Model 2 | 1 | 0.99 | 0.54 | 1.80 | 0.70 | 0.37 | 1.33 | 0.87 | 0.45 | 1.67 | 0.55 |
| Model 3 | 1 | 1.02 | 0.56 | 1.87 | 0.63 | 0.33 | 1.22 | 0.84 | 0.43 | 1.66 | 0.45 |
| Stroke |  |  |  |  |  |  |  |  |  |  |  |
| Model 1 | 1 | 0.77 | 0.46 | 1.28 | 0.97 | 0.60 | 1.57 | 0.75 | 0.44 | 1.27 | 0.40 |
| Model 2 | 1 | 0.77 | 0.46 | 1.28 | 0.95 | 0.59 | 1.55 | 0.73 | 0.43 | 1.26 | 0.36 |
| Model 3 | 1 | 0.72 | 0.42 | 1.23 | 0.97 | 0.59 | 1.60 | 0.74 | 0.42 | 1.32 | 0.44 |
| Hemorrhagic stroke | | | | | | | | | | | |
| Model 1 | 1 | 1.79 | 0.43 | 7.54 | 1.05 | 0.21 | 5.33 | 2.26 | 0.53 | 9.56 | 0.32 |
| Model 2 | 1 | 2.12 | 0.49 | 9.10 | 1.07 | 0.21 | 5.61 | 2.15 | 0.48 | 9.73 | 0.42 |
| Model 3 | 1 | 2.42 | 0.55 | 10.71 | 1.13 | 0.21 | 6.09 | 2.20 | 0.45 | 10.62 | 0.46 |
| Ischemic stroke | |  |  |  |  |  |  |  |  |  |  |
| Model 1 | 1 | 0.67 | 0.39 | 1.17 | 0.96 | 0.58 | 1.59 | 0.62 | 0.35 | 1.10 | 0.19 |
| Model 2 | 1 | 0.66 | 0.38 | 1.16 | 0.94 | 0.56 | 1.57 | 0.61 | 0.34 | 1.10 | 0.18 |
| Model 3 | 1 | 0.59 | 0.33 | 1.07 | 0.96 | 0.57 | 1.62 | 0.60 | 0.32 | 1.14 | 0.23 |

Abbreviation: HR, hazard ratio; CI, confidence interval; py, person year; Q: quartile;

Model 1: adjusted for age and gender,

Model 2: Model 1 & additionally adjusted for body mass index, smoking, drinking, marital status, education level, job, and sports activity,

Model 3: Model 2 & additionally adjusted for hypertension, diabetes, LDL cholesterol and HDL cholesterol level,
